# Supplementary material for: Sero-prevalence and associated risk factors of bovine brucellosis in Sendafa, Oromia Special Zone surrounding Addis Ababa, Ethiopia
Source: PLoS One. 2020 Nov 17;15(11):e0238212. doi: 10.1371/journal.pone.0238212 (PMC7671510; doi:10.1371/journal.pone.0238212)
Supplement: Attachment — Submitted filename: Review PlosOne journal Brucellosis.pdf [file pone.0238212.s002.pdf]

The review of the manuscript entitled "Sero-Prevalence and Associated Risk Factors of Bovine 2 Brucellosis in Sendafa, Oromia Special Zone Surrounding 3 Addis Ababa, Ethiopia" (PONE-D-20-24877) for PLOS ONE and my comments are as follow:

General comments: - The manuscript presents an original work and the study is related to an important zoonotic disease. However, there is a need to improve it in several aspects: the author need to clarify the herd prevalence, within herd prevalence and overall seroprevalence; there are some grammatical errors and should be revised for the improvement;

I recommend being accepted with several revision

2) Methods

-Are the objectives of the study clearly articulated with a clear testable hypothesis stated? In general Yes; I think that drawing the conclusions will be more appropriate by assessing both herd and within herd prevalence.
-Is the study design appropriate to address the stated objectives? It needs some improvement
-Is the population clearly described and appropriate for the hypothesis being tested? In general Yes, however there is a lack of information regarding:

1. Criteria for animals inclusion/exclusion criteria; The sensitivity of diagnostic tests is lower in animals less than one year. Please insert the reference why you tested animals 6 month old? Usually, animals more than one year are tested.
2. Description of any national/regional control program of brucellosis in animals
3. Which criteria were used for declaring a positive herd and or positive animal? Did were attempted to isolate the bacteria from positive animals?
4. Which is the human brucellosis situation in the region?

-Is the sample size sufficient to ensure adequate power to address the hypothesis being tested? The sample size for assessing the prevalence at animal level is sufficient, however it will be more suitable if you use approaches for calculation of sample size in both herd and animal level.
-Were correct statistical analysis used to support conclusions? Formula for sample size calculation is used correctly. You need to calculate herd prevalence and within herd prevalence. Calculation of apparent and true prevalence will be a plus?!!!
-Are there concerns about ethical or regulatory requirements being met? No

3) Results

-Does the analysis presented match the analysis plan? Yes, however, if the author are going to accept the suggestions, there is a need to reflect additional analyze
-Are the results clearly and completely presented? Yes, however there is a need to reflect revisions as consequence of any additional analyzes.

-Are the figures (Tables, Images) of enough quality for clarity? The tables are present at accepted level, however, the manuscript misses the presence of a map of the study region.

4) Conclusions

-Are the conclusions supported by the data presented? Yes
-Are the limitations of analysis clearly described? There are not mention the limitations fully and clearly.

-Do the authors discuss how these data can be helpful to advance our understanding of the topic under study? Yes
-Is public health relevance addressed? Yes

5) Do you have any editorial suggestions or relatively minor modifications of existing data that would enhance clarity? No

6) Please confirm all comments are suitable for the authors to view. If not, please indicate which comments are intended only for the PLOS Editor. Yes, I confirm.

7) Do you have any competing interests? Please describe them below. If you have none, enter "none." None

8) Has all research been conducted to a high ethical standard, and is the work performed the same work which was approved? (Note that PLOS NTDs staff runs an internal ethics check before papers proceed to review, so this is a secondary check.) Yes

9) If you responded “No” to the previous question, please briefly outline your concerns.

10) Have the authors made all data underlying the findings in their manuscript fully available? Yes

11) Is the manuscript presented in an intelligible fashion and written in standard English? Yes, however, English is not my mother language

12) If you responded “No” to the previous question, would additional copyediting make this manuscript acceptable for publication?

13) If accepted, do you think this submission should be press released or receive additional media promotion on the basis of novelty, impact, and/or public interest? Maybe

14) Do you wish to identify yourself to the authors and the other reviewers? (Yes / No) Yes
