# Supplementary material for: Sero-prevalence and associated risk factors of bovine brucellosis in Sendafa, Oromia Special Zone surrounding Addis Ababa, Ethiopia
Source: PLoS One. 2020 Nov 17;15(11):e0238212. doi: 10.1371/journal.pone.0238212 (PMC7671510; doi:10.1371/journal.pone.0238212)
Supplement: Attachment — Submitted filename: Rebuttal Letter.docx [file pone.0238212.s003.docx]

**Dear Publisher**

First of all, we would like to say both **Reviewers** many thanks for their invaluable time and critical and professional evaluation of our manuscript. We have a great appreciation for the corrections given by the reviewers and found them more valuable for the betterment of our manuscript.

When I come to the comments were given by the reviewers’:

**Methods Section:**

1. The comment given by the reviewers related to the inclusion/exclusion criteria of the sampled animals is quite right. We didn’t take any sample from animals below 1 year of age. Hence, I have made the correction as per the comment.
2. Of course, as a professional we had to include the national/regional control program of brucellosis. However, so far there is no any proclamation related to the control program of this disease at regional as well as national level. That is way we didn’t say anything related to it. But, we have indicated it in the conclusion and recommendation section of this manuscript.
3. We didn’t come across for declaring a positive herd as well a positive animal. Moreover, as the title of the manuscript as well as the methodological approach that we used, we didn’t perform any bacteriological as well as molecular characterization techniques.
4. Related to the herd level and within the herd prevalence’s calculation, though as a professional we know such calculations are very crucial, however, we didn’t perform that. Since our country, Ethiopia, didn’t apply the testing and replacement and/or slaughter policies. Moreover, from the beginning of such a research here in Ethiopia, the farm managers and/or owners didn’t want to be notified regarding the status of their farms as well as the herd prevalence of each farm of the study area.

**Result Section:**

1. I have included map of study area as well as the region as per the comments.
